# Uptake and Transformation of Hexachlorocyclohexane Isomers (HCHs) in Tree Growth Rings at a Contaminated Field Site

**DOI:** 10.1021/acs.est.3c01929

**Published:** 2023-06-02

**Authors:** Xiao Liu, Steffen Kümmel, Stefan Trapp, Hans Hermann Richnow

**Affiliations:** † Department of Isotope Biogeochemistry, Helmholtz Centre for Environmental Research-UFZ, Permoserstraße 15, 04318 Leipzig, Germany; ‡ Department of Environmental and Resource Engineering, Technical University of Denmark, Bygningstorvet 115, 2800 Kongens Lyngby, Denmark; § Isodetect GmbH, Deutscher Platz 5b, 04103 Leipzig, Germany

**Keywords:** tree growth rings, bark, isotope fractionation, contaminant transformation, dual-element isotope analysis, phytoscreening

## Abstract

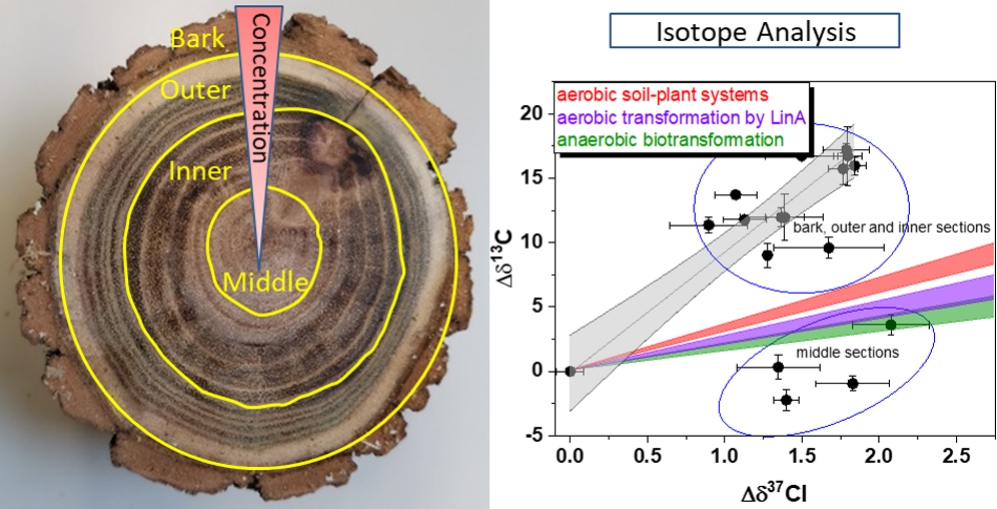

The potential transformation of hexachlorocyclohexane isomers (HCHs) within tree trunks could have a significant impact on the use of phytoscreening. However, the transformation mechanisms of HCH in trunks particularly in growth rings are not yet well understood. Therefore, a field study on an HCH-contaminated field site was conducted to investigate the fate of HCH, particularly α-HCH in tree trunks using multielement compound-specific isotope analysis (ME-CSIA) and enantiomer fractionation. The results indicate that α-HCH was transformed, as evidenced by higher δ^13^C and δ^37^Cl values detected across different growth ring sections and in the bark compared to those in muck and soil. Remarkably, in the middle growth ring section, δ^13^C values of HCH were only marginally higher or comparable to those in muck, whereas δ^37^Cl values were higher than those of the muck, indicating a different transformation mechanism. Moreover, the δ^37^Cl values of β-HCH also increased in the tree trunks compared to those in soil and muck, implying a transformation of β-HCH. Additionally, dual-element isotope analysis revealed that there are different transformation mechanisms between the middle growth rings and other sections. Our findings suggest that the transformation of HCHs in trunks could bias quantitative phytoscreening approaches; however, ME-CISA offers an option to estimate the degradation extent.

## Abstract 2

Elucidation of the transformation of HCH in bark and growth rings of trees at a field site by isotope concepts shed new light on the application of phytoscreening and dendrochemistry.

## Introduction

Hexachlorocyclohexane isomers (HCHs) are classified as persistent organic pollutants (POPs). However, in the industrial synthesis of this compound, the primary product is a mixture of α-, β-, γ-, and δ-HCH. Among the different HCHs, only γ-HCH possesses insecticidal properties. During the purification of lindane (γ-HCH), a large amount of HCH muck (mostly containing α- and β-HCH) was disposed into the environment.^1^ Although HCH was banned in 2009 by the Stockholm Convention,^2^ HCH contamination can still be found in various environmental compartments such as soil,^3^ sediments,^3^ groundwater,^4^ plants,^5^ wild animals,^6^ and even in human blood.^7^ The general microbial transformation pathways of HCH have been identified. In aerobic microbial transformation, the first reaction step of HCH is dehydrochlorination of HCH forming pentachlorocyclohexene, which can be further mineralized,^8^ and in anaerobic pathways, the first reaction step of HCH is dichloroelimination of HCH forming the tetrachlorocyclohexene, which are further dehalogenated and the final products are benzene and chlorobenzene.^8^

Tracing transformation processes of organic contaminants by changes of the concentration alone is challenging since the concentration can be influenced by physical processes such as dilution and sorption in addition to biotransformation. Multi-element compound-specific isotope analysis (ME-CSIA) is a promising concentration-independent approach to assess the transformation of organic pollutants in complex systems.^9−12^ Additionally, enantiomer fractionation could occur during biotransformation processes of chiral compounds, which can be characterized by the enantiomer fraction (EF) and used as an indicator for biological transformation.^13^ Of all HCH isomers, only α-HCH is chiral. Both ME-CSIA and EF have been successfully applied to characterize the biotransformation of HCHs in groundwater, sediment, and soil-plant ecotones.^14,15^ Recently, ME-CSIA and EF were applied to characterize the transformation of HCHs in tree branches, leaves, and fruits sampled at a contaminated site over two annual growth periods, suggesting that transformation varies and changes in transformation pathways in these tree tissues are related to season and climate.^16^

The largest challenge for characterizing dominant transformation pathways under field conditions is that multiple degradation processes (e.g., aerobic and anaerobic or biotic and abiotic degradation processes) can be simultaneously active.^17,18^ However, distinct reaction mechanisms may be identified under field conditions as these mechanisms may involve different modes of chemical bond cleavage with specific isotope effects, which can be identified by ME-CSIA.^15^ Dual-element isotope analysis has already been used to reveal HCH transformation pathways in soil-plant systems^5,14^ and also in trees in a field study.^16^

Phytoscreening/phytomonitoring has become increasingly popular since this method is inexpensive, minimally invasive, and can save time and labor compared to other site characterization methods.^19^ Normally, organic pollutant concentrations in tree cores or branches are examined within phytoscreening to delineate subsurface contamination plumes.^19^ Additionally, it has been proposed as an innovative method to test pollutant concentrations in tree rings to reveal the local history of subsurface contamination.^20^ Contaminants are considered to retain in the annual growth ring formed in the year of uptake. Thus, trees will record the uptake of contaminants over time in dated annual tree rings, referred to as dendrochemistry.^21^ Earlier phytoscreening studies with HCH used bark to identify the direction of the source and the concentration of HCH in the subsurface on a regional or even global scale,^22,23^ which can cover square kilometers with 100s of samples in a single day saving time and labor. However, the potential degradation of HCH and other organic pollutants by trees has a major impact on the interpretation of results from phytoscreening and dendrochemistry. Although degradation in tree trunks may seriously affect the quantitative evaluation of tree ring data, no studies have addressed the potential degradation of HCH and other organic pollutants particularly in the trunks of trees. Previous studies have shown that plants have the potential to transform organic pollutants with enzymes or endophytes. Plant enzymes such as P450 monooxygenases, dehalogenase, glutathione S-transferases (GST), and glucosyl-transferases (UGT) could directly function in the detoxification and deposition of organic pollutants.^24^ The degradation of ibuprofen by plant-derived P450-like enzymes has been reported.^25^ Hybrid poplar (*Populus* spp.), algae (various spp.), and parrot feathers (*Myriophyllum aquaticum*) have been reported to produce dehalogenases, which are able to transform DDT.^26^ The detoxification process of γ-HCH by *Phragmites australis* plants could be attributed to UGT enzymes in the root and the rhizome as well as by GST enzymes in the leaves.^27^ Thus, the objective of the current study was to reveal the fate of HCH in tree trunks. For this purpose, *Robinia pseudoacacia* was selected as test tree species growing on a contaminated test field site (located in Bitterfeld–Wolfen). This species was selected as it is one of the most abundant trees in the study area. The uptake and transformation of HCHs was analyzed based on changes in concentration and isotopic composition in different parts of tree trunks (i.e., outer, inner, and middle section of tree growth rings and bark samples). Dual-element isotope analysis was applied to identify the different HCH transformation mechanisms in the different parts of the trunks.

## Methods and Materials

### Field Site

The sampling site is located in Bitterfeld–Wolfen (51.6395°, 12.2880°) and belongs to the core center of the chlorine chemistry of the former German Democratic Republic. This area was one of the most HCH-contaminated sites in the world.^28^ The studied area was a former loading site for HCH waste. The concentration gradient of HCH in the soil is very high in the loading areas with pure white crystals and gray muck (sludge of the synthesis from HCH) to only a few mg kg^–1^. It is hard to predict an average concentration for the rhizosphere of the trees because the distribution of muck in the subsurface is unknown. Today, the area is covered by a vegetation of bushes and trees. More details can be found in S1 (Supporting Information (SI)) and a former publication.^16^

### Sampling of Trees

All samples were collected between November 2019 and December 2022. Trunk cuttings from different *R. pseudoacacia* trees were taken to monitor concentrations and isotopic compositions of HCHs. *R. pseudoacacia* is a pioneer species and one of the most abundant trees in the vegetative succession on highly contaminated soils in this area. The selected trees were more than ten years old. In total, four trunks of different *R. pseudoacacia* were sampled: in November 2019 (1), in March 2021 (2), and in December 2022 (1). Based on the sampling time, we numbered the different trunks from 1 to 4, such as T-1 (trunk 1). In close vicinity of the sampled *R. pseudoacacia*, HCH muck (mostly consisting of pure α- and β-HCH) could be found at the upper sections of the soil (0–20 cm). The trunk cuttings of *R. pseudoacacia* were divided into different sections based on the tree rings, as shown in Figure S1. From the outside to the middle of the trunk, up to 4–5 circles of tree rings were defined as one sample. Each sample was collected by drilling through the trunk as shown in Figure S2. Therefore, one trunk consists of samples including bark, outer, inner, and middle growth rings. The growth year of tree rings and bark were counted as follows: the bark was counted as number 0, and from the bark dated back to the middle rings of trees, one growth ring counted as one year (age 1). For example, T-1 contains the bark (0), outer (1–5), inner (6–9), and middle (10–13) sections of growth rings. The details can be found in Table S1. All of the samples were stored at −20 °C until further treatment.

### Extraction and Cleanup of HCHs

A detailed description of the extraction and cleanup procedure of HCHs from plant samples is described elsewhere.^29^ The extraction and cleanup methods used did not have any influence on the carbon and chlorine isotopic compositions of HCH.^29^ Further details are summarized in S2 (SI).

### Analytical Methods

#### Concentration Analysis

An Agilent 6890 series GC (Agilent Technologies, USA) equipped with a flame ionization detector (FID) was used to determine the concentration of HCHs throughout the study. Further analytical details are documented in S3 (SI).

#### Isotope Analysis

The isotopic composition of an element (*E*) was reported as δ notation in parts per thousand (‰) according to eq 1.
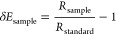
1where *R*_sample_ and *R*_standard_ are the isotopic ratio of the sample and an international reference standard for the element of interest (e.g., Vienna PeeDee Belemnite (V-PDB) for carbon and Standard Mean Ocean Chloride (SMOC) for chlorine).

#### Carbon Isotope Analysis

Carbon isotopic compositions (δ^13^C) were analyzed by gas chromatograph-combustion-isotope ratio mass spectrometry (GC-C-IRMS), where a GC (7890A, Agilent Technologies) was connected through a GC-IsoLink and a ConFlo IV interface to a MAT 253 IRMS system (Thermo Fisher Scientific, Germany). Further analytical details are documented in S3 (SI).

#### Chlorine Isotope Analysis

Chlorine isotopic compositions (δ^37^Cl) were analyzed using a gas chromatograph (Trace 1310, Thermo Fisher Scientific, Germany) coupled with multiple-collector inductively coupled plasma mass spectrometry (GC-MC-ICPMS; Neptune, Thermo Fisher Scientific, Germany), as recently described elsewhere.^30^ Further analytical details are documented in S3 (SI).

#### Dual-Element Isotope Analysis

The lambda (Λ) value was used to distinguish different transformation mechanisms in complex systems.^5^ Λ is defined as the slope of the regression line of the isotope fractionation of two elements during transformation processes, e.g., due to microbial transformation or transformation reactions related to plant tissues. Therefore, δ^13^C and δ^37^C values of HCH detected in tree rings were normalized and compared to the HCH source, which is represented by the HCH muck in the present study.

#### Enantiomer Analysis

The enantiomer fraction (EF) (−) is defined as A–/(A+ + A−), where A+ and A– correspond to the peak area or concentrations of (+) and (−) enantiomers. However, the photochemical synthesis of HCH results in a racemic mixture of α-HCH (EF (−) = 0.5), which is typically found in the HCH muck. The EF (−) was analyzed by gas chromatography-mass spectrometry (GC-MS) (Agilent Technologies 7890A for GC and 5975C for MS) equipped with a γ-DEX 120 chiral column (30 m × 0.25 mm × 0.25 μm, Supelco, Bellefonte, PA). Further analytical details are summarized in S3 (SI).

#### Calculation of the Extent of Degradation (% Biodegradation) and Reconstruction of the Initial Concentration

For the quantification of α-HCH transformation by isotope analysis, the simplified Rayleigh equation was applied for calculation of biodegradation (B%), as shown in eq 2. δ_0_ is the initial or referential carbon isotope value of the whole study, and in the current study, δ_0_ represents the carbon and chlorine isotope value of HCH muck with a value of −27.2‰ and −1.9‰, respectively. Δδ is the difference of carbon or chlorine isotope value of tree trunk samples between the HCH muck. ε_c_/ε_cl_ is the carbon or chlorine isotope fractionation factor.
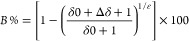
2

The reconstructed concentration (RC) was calculated using the percentages of biodegradation and the residual concentration in different sections, which is shown in eq 3.
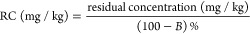
3

#### Statistical Analysis

The HCH concentration, isotope, and enantiomer data were analyzed statistically using analysis of variance (ANOVA) and Duncan post hoc comparison testing with the SPSS software v19.0.

### Quality Assurance and Quality Control

Strict quality control criteria were complied within all analytical procedures to ensure correct identification and quantification of HCHs. A linearity check prior to the carbon and chlorine isotope analyses was performed using HCH standards for checking the linear range where the isotope values are independent of the concentrations of HCH. Moreover, a calibration using in-house standards was performed for chlorine isotope analysis for ensuring the stability of the systems. To ensure the reproducibility of the isotope composition of HCH, the known isotope values of in-house HCH standards were injected after every 3 samples. To ensure enantiomeric analysis, racemic α-HCH standards were injected after every 3 samples. Only when the EF (−) values of α-HCH standards were in the range of 0.496–0.504 (95% confidence interval), the measured data of the samples in the same sequence were accepted. The calculated limits of detection (LOD) values of HCHs were 0.5–1 nmol C and 1–2 nmol Cl injected on the column for carbon and chlorine isotope analysis, respectively. In the current study, all of the δ^13^C and δ^37^C values of HCH detected in tree rings were compared to the HCH source, which is represented by the HCH muck in the present study. The correlation line was performed by fitting with X error using the software Origin.

## Results and Discussion

### Uptake and Translocation of HCH

**Figure 1:**
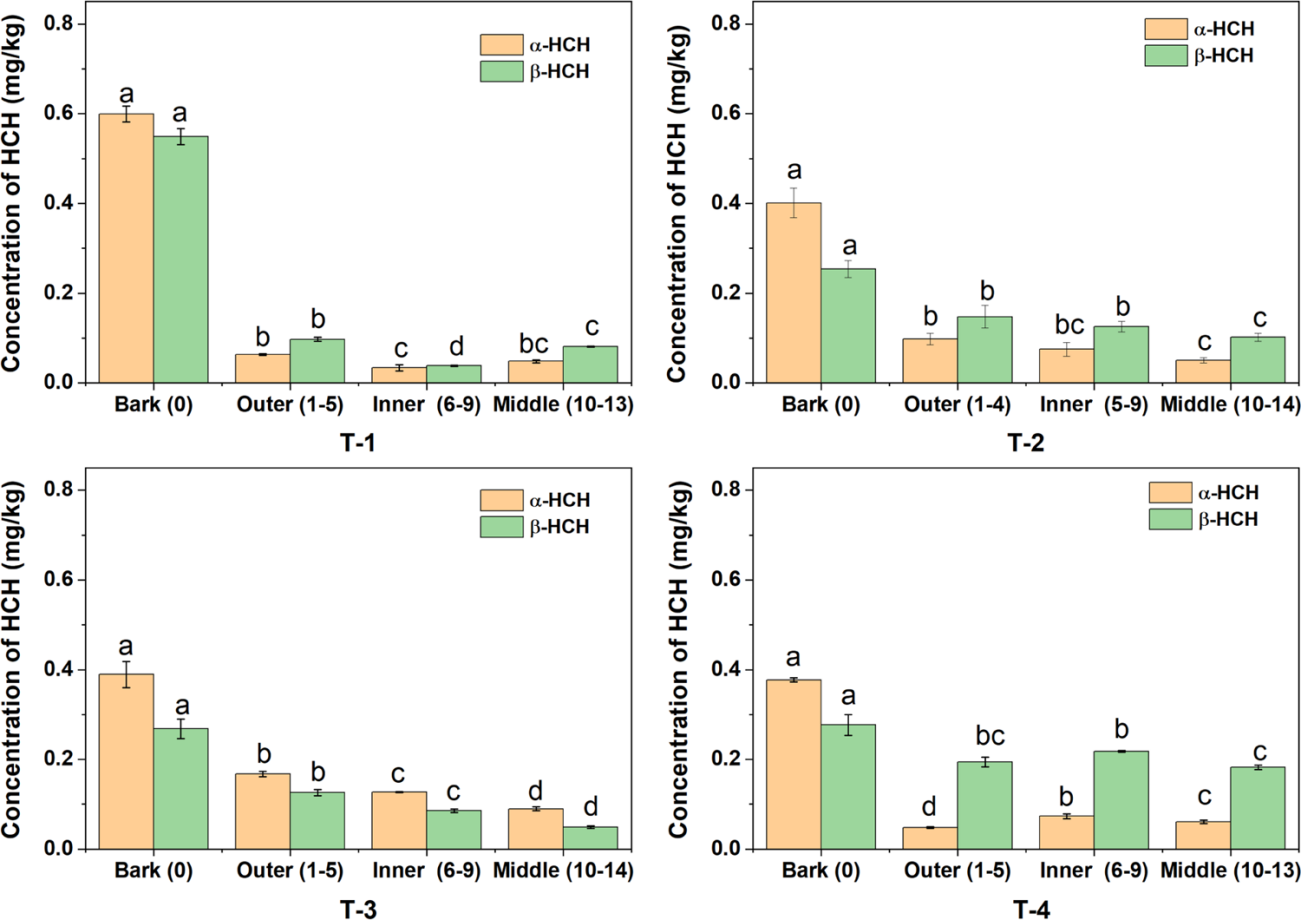
Concentration of α- and β-HCH in different parts of tree trunks including the bark, outer, inner, and middle growth tree rings of *R. pseudoacacia*. The numbers at the x-axis represent the counted tree growth rings from bark (0) dated back to the middle section. The letters a–d in all figures represent statistically significant differences between different tree ring sections according to Duncan’s test (*p* < 0.05).

Figure 1 shows the distribution of α- and β-HCH in different parts of the investigated tree trunks including bark, outer, inner, and middle tree rings of *R. pseudoacacia*, indicating the tree uptake of HCH from the source and translocation within the trees. Gray and white materials were found in the soil at the Bitterfeld–Wolfen sampling site. The so-called HCH muck was deposited here in a former landfill and therefore can be considered as the source of contamination in this area. Generally, the uptake and accumulation of HCHs by plants except bark is hypothesized to occur through two pathways: (a) soil-to-plant translocation by root uptake and (b) air-to-plant translocation mainly by leaf uptake.^31−33^ As demonstrated previously for the Bitterfeld–Wolfen site,^14^ leaf uptake accounts for only a very small proportion compared to root uptake. Accordingly, uptake via leaves can be neglected as a potential uptake pathway, so that the main route of HCH uptake into tree trunks is via the roots. However, the root uptake is mainly driven by the water flux, which is governed by evapotranspiration. Water movement in trees is passive, flowing from regions of high-water potential in the roots to regions of low-water potential in the leaves.^34^ The outside of the trunk is made of bark and the underlying phloem. The majority of water flows axially upward within the xylem tissue in the sapwood in the outer, young tree rings. However, the uptake of HCH from air by the bark at the contaminated field site is also a main process besides root uptake. Uptake of hydrophobic compounds into the bark from air has been shown at contaminated sites^35^ and also at a global scale.^36^

As shown in Figure 1, the highest HCH concentrations of all tree trunks were observed in the bark ranging from 0.38 ± 0.00 to 0.60 ± 0.02 mg/kg for α-HCH and 0.28 ± 0.00 to 0.55 ± 0.02 mg/kg for β-HCH, respectively. HCH is a hydrophobic substance and thus is not actively taken up by plants.^37^ Bark consists to a high degree of suberin, a highly lipophilic polymer with excellent capabilities to adsorb hydrophobic compounds from air, which explains why bark has the highest HCH concentrations. Adsorption of HCHs to the bark may also occur from the phloem or xylem flowing inside the bast and wood, but it is not possible to discriminate between these pathways. Consistent with previous studies, bark samples exhibited higher concentrations of α-HCH relative to β-HCH.^23,38^ This can be attributed to the ability of the bark to act as a passive sampler and thus enabling the uptake of HCH from air, and particularly it enriches α-HCH over the other HCH isomers.^23,38^ The vapor pressure of α-HCH is higher than that of β-HCH,^39^ and more α-HCH may evaporate from the soil, which may subsequently lead to high accumulation of α-HCH in the bark. However, there is no systematic higher concentration of α-HCH in the bark. In a previous study, β-HCH in the bark showed two times higher concentrations than α-HCH,^22^ and in another study, α-HCH was more than five times higher than β-HCH.^23^ Thus, the site-specific (or regional) conditions, e.g., the composition of HCHs in the source, concentration in the air, and temperature are the major factors governing the accumulation of HCH in the bark via the gas phase. A decrease of the HCH concentration from the bark to the middle of the trunk growth rings was observed in all tree trunks and the concentration of α- and β-HCH in outer, inner, and middle growth rings of trunks ranged from 0.05 to 0.17 and 0.05 to 0.22 mg/kg, respectively (Figure 1). Compared to bark, wood contains a lower proportion of suberin and a higher proportion of lignin and cellulose, which are less effective sorbents for hydrophobic compounds.^33^

The outer, inner, and middle sections of trunk growth rings showed higher concentrations of β-HCH than α-HCH (Figure 1) indicating either a lower extent of β-HCH degradation or a more effective accumulation of β-HCH in these parts. The latter aligns with the higher hydrophobic nature of β-HCH, specifically its higher K_ow_ values,^33^ leading to its stronger affinity to adsorb to lignin domains of the wood. However, the relative enrichment of β-HCH in these sections may also indicate a lower transformation, given its persistence relative to α-HCH. Nevertheless, this issue cannot be determined solely based on concentration levels.

### Carbon and Chlorine Isotope Fractionation of HCHs

**Figure 2:**
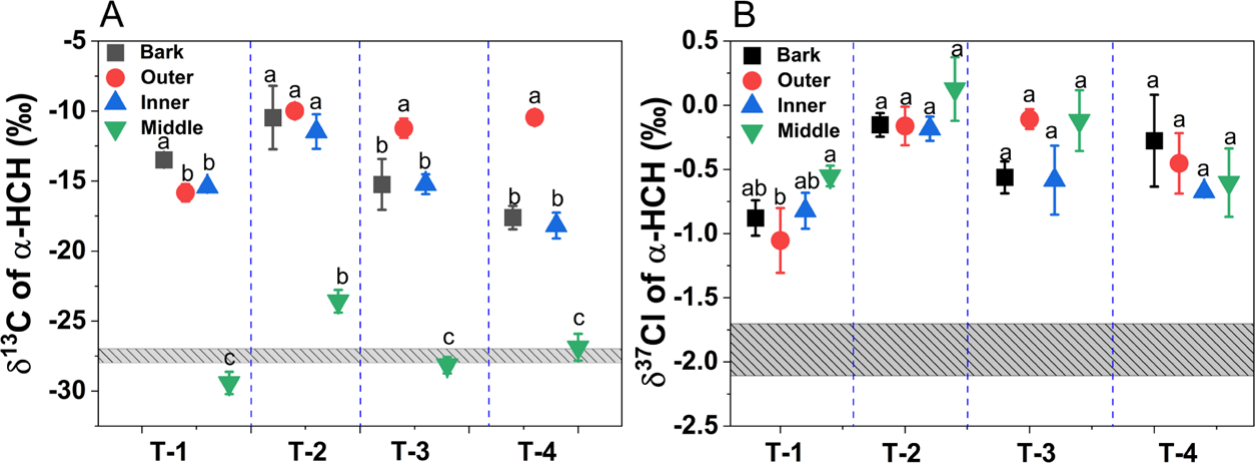
δ^13^C and δ^37^Cl values of α-HCH in different parts of tree trunks including the bark, outer, inner, and middle section of growth tree rings of *R. pseudoacacia*. The gray bar in panel A represents the carbon isotopic composition of α-HCH detected in the HCH muck. The gray bar in panel B represents the chlorine isotopic composition of α-HCH detected in the HCH muck. The letters a–d in all figures represent statistically significant differences between different tree ring sections according to Duncan’s test (*p* < 0.05).

Both carbon and chlorine isotopic compositions of α- and β-HCH were analyzed to investigate the transformation of HCHs in tree growth rings. However, due to a peak overlap of β-HCH with nonchlorinated compounds, only δ^37^Cl values could be obtained for β-HCH, while δ^13^C values could not be acquired. The average δ^13^C and δ^37^Cl values of α-HCH in the muck were found to be −27.2 ± 0.3 and −1.9 ± 0.2‰, respectively. The average δ^37^Cl values of β-HCH in the muck were −2.8 ± 0.2‰. The isotope values of HCH muck exhibit low variability.^16^ Similar δ^13^C and δ^37^Cl values of HCH between soil and muck in this area were identified in a previous study^16^ and muck also presents in surface soil, which represents the predominating source of HCHs. For further evaluation, all isotope values of HCHs detected in tree trunks were compared to those detected in the muck. Notably, in all trunk samples (except for the middle section) the δ^13^C and δ^37^Cl values of α-HCH were much higher than those of the muck. A difference of +8.7 to +17.2‰ for carbon and +0.9 to +1.8‰ for chlorine was observed (Figure 2), suggesting a transformation involving the cleavage of a C–Cl bond of α-HCH in the trunks. The range of δ^13^C and δ^37^Cl values of HCHs in the bark, outer, and inner growth ring sections varied from −10.0 ± 0.5 to −18.5 ± 1.5‰ for carbon and −0.1 ± 0.1 to −1.1 ± 0.3‰ for chlorine, respectively (Figure 2), and thus indicate that the extent of α-HCH transformation mediated by the plant differs across various growth ring sections. This variability could potentially be linked to the specific activities of plant enzymes or endophytes in different stages of tree growth. With the exception of the bark, the δ^13^C and δ^37^Cl values of α-HCH followed a general trend, where the inner growth rings of the trunks exhibit lower values compared to the outer growth rings. This indicates that there is a gradual transformation from older to younger tree growth rings related to the growth ages of trees. The δ^13^C values of HCH in the middle section, which is typically composed of dead wood and considered the oldest part of the tree trunk, show a distinct difference from other compartments of the trunk and are similar to the values observed in the muck (Figure 2). On the other hand, the δ^37^Cl values of HCH in the middle section exhibit the highest values across all samples. This suggests that there is a substantial chlorine isotope fractionation but only a small to almost negligible carbon isotope fractionation in the middle stem. This stands in contrast to the other sections of the tree trunk, which showed both carbon and chlorine isotope fractionation. Based on these results, it can be hypothesized that a specific transformation pathway, with small or negligible carbon isotope fractionation in the rate-limiting step, was dominant in the middle rings of trunks. This indicates the existence of distinct processes that are unique for the older and dead middle wood and may be related to a specific community of endophytes inhabiting that region. It is known from previous research on various environmental compartments that aerobic and anaerobic biotransformation as well as abiotic transformation can result in differing degrees of carbon and chlorine isotope fractionation of HCHs.^39^ Hence, the growth time, composition, and conditions across different growth rings may lead to alterations in the community of degrading endophytes (originating from trees or inoculating by soil bacteria) or enzymes, subsequently leading to differences in transformation extents and mechanisms. In addition, the variability in transformation extents and mechanisms in the trunks suggests that there is no integral component, which can adequately represent the overall isotope change or transformation/concentration shifts. This has significant implications for tree core studies.

**Figure 3:**
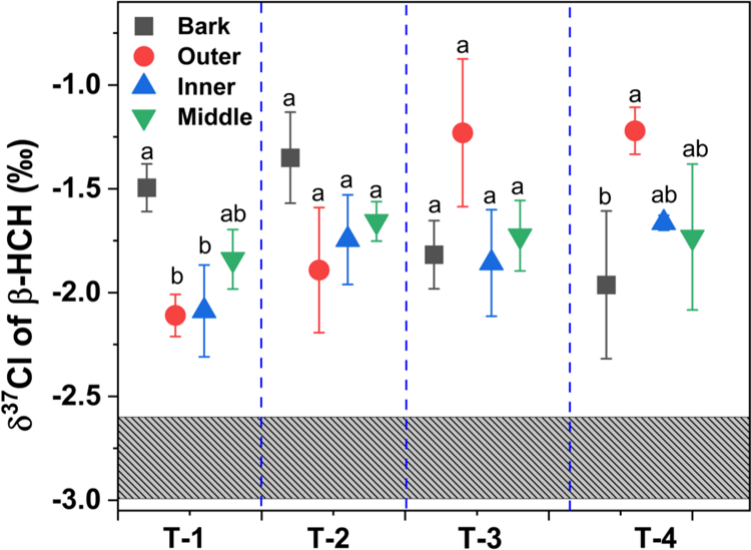
δ^37^Cl values of β-HCH in different parts of tree trunks including the bark, outer, inner, and middle sections of growth tree rings of *R. pseudoacacia*. The gray bar represents the chlorine isotopic composition of β-HCH detected in the HCH muck. The letters a–d in all figures represent statistically significant differences between different tree ring sections according to Duncan’s test (*p* < 0.05).

The δ^37^Cl values of β-HCH were also determined, but it was not possible to analyze the δ^13^C values of β-HCH due to peak overlap, as explained above. Similar to the case for α-HCH, the δ^37^Cl values of β-HCH in all samples were higher than those observed in the muck (Figure 3), signifying the transformation of β-HCH within the tree trunks and the chlorine bond cleavage being involved in the rate-limiting step. Prior studies have demonstrated that the transformation of β-HCH is primarily driven by soil bacteria inoculation, which subsequently becomes plant endophytes that facilitate the transformation.^5^ The observation of β-HCH transformation within trunks at the contaminated site was consistent with the former study.^16^ However, due to the unavailability of δ^13^C values for β-HCH, it is challenging to distinguish between transformation pathways in the middle tree rings compared to other sections, as it was observed for α-HCH. Additionally, compared to the α-HCH concentration, usually higher β-HCH concentrations were observed in the outer, inner, and middle growth rings. Based on the δ^37^Cl values of β-HCH, it can be ruled out that the higher β-HCH concentrations were related to lower degradation extents. Therefore, it can be suggested that the increased β-HCH concentrations in the outer, inner, and middle growth rings are most likely associated to the physiochemical properties of β-HCH, specifically its higher K_ow_ values,^33,35^ which is correlated to adsorption to lignin,^40^ resulting in higher accumulation of β-HCH in these woody sections, even when transformation potentially affects concentrations.

### Enantiomer Fractionation of α-HCH

**Figure 4:**
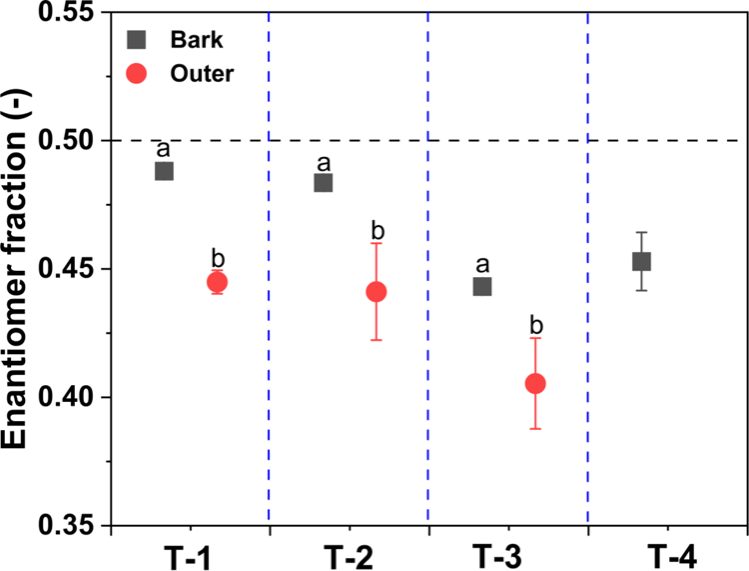
Enantiomer fractionation of α-HCH in different parts of tree trunks including the bark, outer, inner, and middle sections of growth rings of *R. pseudoacacia*. The black dashed line represents the enantiomer fraction (EF(-)) detected in the HCH muck. The letters a–d in all figures represent statistically significant differences between different tree ring sections according to Duncan’s test (*p* < 0.05).

Enantiomer fraction (EF) occurs during the biotransformation of chiral compounds and can thus serve as an indicator of biotransformation.^40^ Additionally, enantiomer fractionation of chiral compounds can theoretically also be caused by plant uptake from the soil or gas phase and the translocation of compounds in the plant.^41^ This was not yet specifically studied for α-HCH. Because of the low α-HCH concentration in the inner and center growth rings, only the bark and the outer growth rings could be analyzed for EF values of α-HCH. Notably, all EF (−) values were lower than those of the muck, which has a racemic composition (EF (−) value = 0.5) (Figure 4). This suggested a preferential transformation of (−) α-HCH or a preferential accumulation of (+) α-HCH by uptake in the bark and the outer growth rings, which is consistent with previous studies.^22,42^ Moreover, the lower EF (−) value of the outer growth rings (representing the youngest growth rings) compared to that in the bark indicated a higher transformation of (−) α-HCH or a higher accumulation of (+) α-HCH, which is partially consistent with the results of the stable isotope analysis. Complementary to the isotope fractionation, the enantiomer fractionation confirmed that the biotransformation of α-HCH occurred faster in the wood of the trunks than in the bark. To the best of our knowledge, enantiomer fractionation of HCH in microbial cultures is only observed under aerobic conditions, as shown in a previous study.^43^ Therefore, it is likely that the transformation of HCH in the bark and the outer growth rings occurred under aerobic conditions.

### Dual-Isotope Analysis for Characterizing HCH Transformations

**Figure 5:**
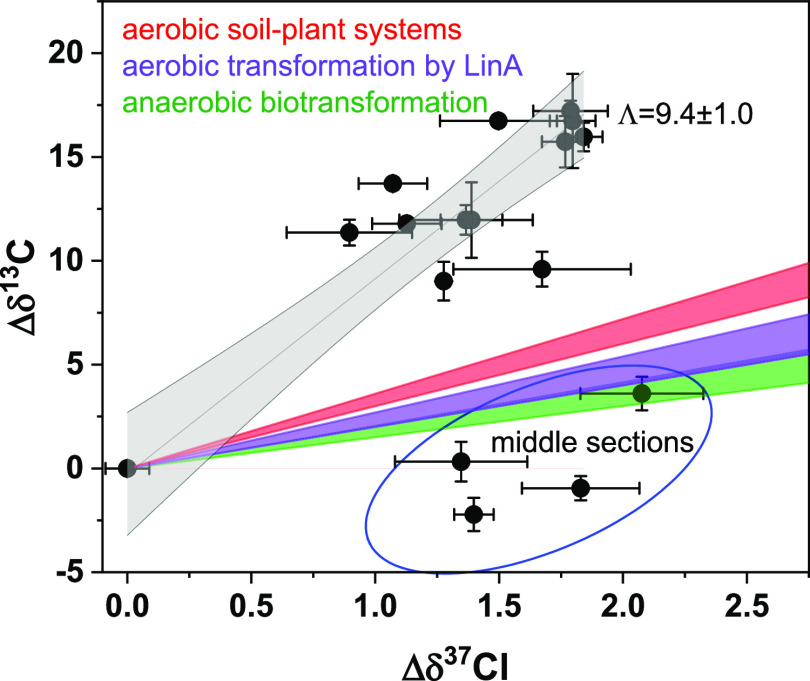
Dual-element isotope analysis (C–Cl) of α-HCH detected in different parts of tree trunks including the bark, outer, inner, and middle sections of growth rings of *R. pseudoacacia*. The purple area represents the Λ values obtained from the aerobic transformation of α-HCH by LinA enzymes.^44^ The green area represents the Λ values obtained by the anaerobic microbial transformation of α-HCH.^44^ The red area represents the Λ value obtained by the aerobic transformation of α-HCH in soil-plant systems.^14^ The samples inside the blue cycle are the center samples of tree growth rings. The gray line represents the correlation line between carbon and chlorine isotope fractionation of tree trunk samples (except the middle section) and the gray area represents the confidential bonds at level 95%.

Dual-element isotope analysis (δ^13^C vs δ^37^Cl) was conducted to investigate the reaction pathways involved in the transformation of HCH in tree growth rings. The obtained results were then compared to previous Λ values acquired from aerobic α-HCH transformation by LinA enzymes (2.4 ± 0.4 to 2.5 ± 0.2),^44^ anaerobic α-HCH transformation (1.7 ± 0.2 to 2.0 ± 0.3),^39^ and aerobic α-HCH transformation in soil-plant systems (3.3 ± 0.3).^14^ Among all middle ring samples, only one sample from T-1 was within the range of anaerobic microbial transformation. The rest of the middle ring results were clustered together and were slightly outside the range of anaerobic microbial transformation (Figure 5). One hypothesis to explain these findings is that the transformation of α-HCH in the middle ring section may be at least partly influenced by anaerobic pathways. The anaerobic conditions in the middle section may be due to the longer distance reducing the O_2_ diffusion causing O_2_-limited conditions and also related to the old, dead, and possibly decaying wood, which may have caused anaerobic microenvironments facilitating anaerobic transformation.^45^ All other samples did not fall within the known processes of α-HCH transformation and displayed high carbon isotope fractionation, indicating that the main pathways in tree trunks are not the mentioned reference pathways. Moreover, all samples, except those from the middle section, displayed a linear regression slope (Λ) of 9.4 ± 1.0 (Figure 5), suggesting that the overall transformation process of α-HCH in the trunks is related to a similar mode of C–Cl bond cleavage. A previous study conducted at the same contaminated field site revealed that the transformation of α-HCH in the same species of tree can vary throughout the annual growth period.^16^ Furthermore, during the same sampling period, the upper parts of the tree (e.g., branches, leaves, and fruits) exhibited a transformation mechanism characterized by high carbon isotope and low chlorine isotope fractionation.^16^ Accordingly, the results of the current study align with previous research and indicate the existence of a dominant transformation pathway throughout the entire tree after the uptake of HCH into the tree, followed by its subsequent translocation into the upper tree parts. The low chlorine and high carbon fractionation can be attributed to a preferential cleavage of a C–H bond in the rate-limiting step, which could be related to a dehydrochlorination.^4,46^ Modeling studies of γ-HCH dehydrochlorination also support this assumption, as experiments have shown that the chlorine fractionation may be low with Λ (C–Cl) values ranging from 3.5 to 5.2 as the initial rate-limiting step is a C–H bond cleavage.^47^

### Estimated Potential Transformation of HCH in Tree Growth Rings

The isotopic compositions observed for HCH residues in tree growth rings indicate that the HCH has undergone transformation processes within the tree trunk. Therefore, it can be assumed that the total amount of HCH taken up into the tree was higher than that indicated by the measured residual concentration. While ^13^C and ^37^Cl analyses do not allow a precise prediction of the HCH transformation pathway in tree growth rings, the Rayleigh equation can be employed to estimate the extent of HCH transformation by utilizing isotope fractionation factors (ε) determined in laboratory experiments (see S4, eq 1). The ε values for aerobic and anaerobic transformation could be used to estimate the order of transformation using the Rayleigh equation. The ε_C_ and ε_Cl_ values for aerobic (ranging from ε_C_ = −1.0 ± 0.2 to −1.6 ± 0.3‰ for microbial cultures^48^ and ε_C_(+)α-HCH = −10.8 ± 1.0‰, ε_C_(−)α-HCH = −4.1 ± 0.7‰, ε_C_(+)α-HCH = −4.2 ± 0.5‰, ε_Cl_(−)α-HCH = −1.6 ± 0.2‰ for LinA enzymes^44^) and anaerobic transformation (ranging from ε_C_ = −2.4 ± 0.2 to −4.2 ± 0.4‰ and ε_Cl_ = −1.4 ± 0.3 to −2.0 ± 0.3‰^44^ for microbial cultures) were compiled to calculate the transformation extent. This method enables a rough estimation of the initial HCH uptake using the Rayleigh equation, which is described in detail in the Methods section (eq 2).

To calculate the extent of HCH transformation, the HCH muck was considered as the source and its isotopic composition represents the initial isotopic composition of HCH taken up by the trees (Methods section, eq 3). The calculation assumes that at least the uptake in the inner sections took place during growth. If a continuous flux of HCH from the root zone in the trunk provides a continuous addition of HCH with an isotope value of the source, the real degradation might be larger and the calculated percent of biodegradation (*B*%) represent the minimum transformation needed to explain the enrichment of heavy isotopes.

As a result, the δ^13^C values of HCH detected in the trunk samples (except the middle growth rings) changed by +9.0 to +17.2‰, indicating a reduction ranging from 54.3 to 99.3% (LinA enzymes) or 86.6 to 99.9% (microbial cultures) of the initial α-HCH concentration using ε_C_ values for aerobic conditions, and a reduction ranging from 86.6 to 99.9% of the initial α-HCH concentration using ε_C_ values for anaerobic conditions (Table S2). In contrast, in the middle rings, the δ^13^C values shifted by 0.3 to 3.6% compared to the HCH muck suggesting a reduction of 2.7 to 66.3% (LinA enzymes), 16.1 to 99.0% (aerobic microbial cultures), or 7.0 to 81.4% (anaerobic microbial cultures) of the initial α-HCH concentration (Table S2). The large variability of reduction of HCH is related to that one sample in the middle sections falls into the anaerobic pathways and the isotope values of others are similar to those of HCH muck. Accordingly, based on the ε_C_ values, the transformation extent in the middle rings was lower compared to the other sections of the tree trunks. However, on examining the δ^37^Cl values, a change ranging from 0.9 to 2.1‰ was observed in all, which corresponds to a transformation extent ranging from 17.4 to 77.3% (LinA enzymes) or 32.3 to 84.8% (anaerobic microbial cultures), respectively (Table S2). Thus, a similar transformation extent was noted in the middle rings compared to the other sections of the tree trunks if ε_Cl_ values were used for the calculation. When applying a low fractionation factor (ε) such as those published for aerobic pathways, the calculated *B*% exceeds 99.9%, which may be questionable for a realistic assessment, and oversimplified assumptions were used for the calculation of a very high degree of degradation. For example, the assumption that the muck represents the source with representative isotope composition is questionable if degradation in the soil already leads to significant enrichment of heavy isotopes or a slow flow of degraded HCH within the trunk, which is subject to continuous degradation prior to deposition in the trunk. However, most importantly, the reliability and accuracy of this estimation are highly dependent on selecting appropriate isotope fractionation factors, and it was found that the transformation mechanism in the middle rings differed from those of the other tree trunk sections, resulting in varying transformation extents when using ε_C_ and ε_Cl_ values. Therefore, to obtain more precise and accurate estimations in future, reference experiments involving tree endophytes or enzymes will be needed to determine appropriate isotope fractionation factors for analyzing transformation segments of tree growth rings. Additionally, as mentioned above, it may be also essential to distinguish the relative contribution of transformation of HCH in the tree between soil and tree itself.

### Reconstructed Concentrations in Tree Growth Rings

Assuming that degradation affects the concentration in tree trunks, transformation extents and residual concentrations can be used to derive biodegradation scenarios based on fractionation factors found in previous studies (see above). Calculation specifics are summarized in the Methods section, and Table S3 displays the reconstructed concentrations with the assumption that HCH with an isotope signature of the muck were adsorbed in tree rings during growth and biodegradation changing the isotope composition. It is further assumed that no significant transport in the middle and inner trunk section from below takes place. In case transport of HCH from the root zone takes place, the calculated values show the minimum extent of degradation as the observed isotope composition could be affected by HCH with the signature on the muck. The reconstructed concentrations which were calculated based on ε_C_ and ε_Cl_ values indicated higher concentrations (Table S3), potentially reflecting actual exposure and being advantageous for phytoscreening purposes. Nevertheless, it is important to note that fractionation factors are currently only available to a limited extent and solely represent microbial degradation and transformation via LinA enzymes. Therefore, reconstructed concentrations should be interpreted with care. However, reconstruction concentrations reveal that tree trunks can accumulate and degrade HCH. Notably, the reconstructed concentrations are significantly higher than the solubility of HCH in water. Assuming that uptake is regulated by the water cycle in the tree, this indicates that HCH may accumulate in the tree trunks due to sorption processes, followed by its in situ degradation making residual concentrations detected in tree trunks less valuable for reconstructing exposure and contamination history. Additionally, it should be noted that this back-calculation assumes that there were no other fractionation processes happening within the tree trunks that have yet to be discovered.

### Environmental Implications

Compared to the previous study about the transformation of HCH in trees over two annual growth cycles,^16^ the current study goes beyond the previous one in which we examined the transformation of HCHs in tree growth rings and focused on the discussion of phytoscreening, which had not been previously investigated. This work is directly related to phytoscreening and the previous study stated that concentration data of organic contaminants for phytoscreening/phytomonitoring in a quantitative way should be taken with caution.^16^ In the current study, we provide the evidence to explain this in detail. The transformation extent of both α- and β-HCH were observed in various sections of tree trunks such as the bark, outer, inner, and middle growth rings. Moreover, α-HCH in middle growth rings followed a different transformation pathway compared to other sections of the tree trunk. By using the Rayleigh equation, the HCH degradation extent could be estimated, which ranged from 54% up to 99.9%. Additionally, isotope analysis provided valuable evidence for the assessment of HCH uptake and transformation in tree trunks, where an assessment solely based on concentration is challenging. Furthermore, dual-element CSIA and enantiomer fractionation analysis allow for revealing and distinguishing transformation processes in field studies.

Earlier phytoscreening studies with HCH and other persistent organic pollutants used the bark to identify the direction of the source on a regional or even global scale.^32^ Here, we show that phytoscreening using sampling of inner wood can be used to screen for HCH sources in soil on a local scale, thereby reducing costs and the time involved in contaminated site characterization.^19^ However, our research indicates that highly degraded fractions are found in the trunk. This implies that concentration values alone from phytoscreening need to be taken with reservation as they do not necessarily reflect the situation in the soil and upper aquifer. Therefore, the current study has implications for the phytoscreening of HCH by tree cores, and in the future, the transformation of HCH in tree rings should be taken into consideration for the calculation of real exposure. Additionally, future work should also focus on characterizing the detailed mechanisms of the transformation of HCH in different sections of tree growth rings, which was not covered in the current study.

## Supporting Information Available

Details on field description, extraction and cleanup methods, analytical methods, transformation calculation, and sampling preparation of tree trunks (PDF)
