# Supplementary material for: Uptake and Transformation of Hexachlorocyclohexane Isomers (HCHs) in Tree Growth Rings at a Contaminated Field Site
Source: Environ Sci Technol. 2023 Jun 2;57(23):8776–84. doi: 10.1021/acs.est.3c01929 (PMC10269325; doi:10.1021/acs.est.3c01929)
Supplement: Supplementary file 1 — es3c01929_si_001.pdf [file es3c01929_si_001.pdf]

Supporting Information

Uptake and Transformation of Hexachlorocyclohexane Isomers (HCHs) in Tree Growth Rings at a Contaminated Field Site

Xiao Liu *[†], Steffen Kümmel [†], Stefan Trapp [‡], Hans Hermann Richnow*[†, §]

[†] Department of Isotope Biogeochemistry, Helmholtz Centre for Environmental Research-UFZ, Permoserstraße 15, 04318 Leipzig, Germany.

[‡] Department of Environmental and Resource Engineering, Technical University of Denmark, Bygningstorvet 115, 2800 Kongens Lyngby, Denmark.

[§]Isodetect GmbH, Deutscher Platz 5b, 04103 Leipzig.

*Corresponding Authors

Department of Isotope Biogeochemistry, Helmholtz Centre for Environmental Research-UFZ, Permoserstraße 15, 04318 Leipzig, Germany

Tel: +49-3412351018; Email: xiao.liu@ufz.de

Department of Isotope Biogeochemistry, Helmholtz Centre for Environmental Research-UFZ, Permoserstraße 15, 04318 Leipzig, Germany & Isodetect GmbH, Deutscher Platz 5b, 04103 Leipzig, Germany

Tel: +49(0)17662660371; Email: hans.richnow@ufz.de; richnow@isodetect.de

The supporting information contains: 12 pages, 2 table, 2 figures.

S1 Field site

The site is located in Bitterfeld-Wolfen and belongs to the core center of the chlorine chemistry of the former German Democratic Republic. This area belongs to the most heavily HCH-contaminated sites in the world [1]. Beside the industrial manufacture of about 4500 chlorine-based chemical substances or associated consumer goods, HCH and DDT were extensively synthesized in Bitterfeld-Wolfen between 1951− 1982 [2,3]. Open-pit mines were used in this period to dump chemical waste from industrial production without any appropriate safety or environmental protection measures. Today most of the contaminated soil in the production areas has been remediated or stabilized. However, the former loading areas next to one previous factory still have some hot spots of soils heavily contaminated by muck and now covered by a vegetation of bushes and trees with an estimated age of more than 20 years. The trees selected for the present study were growing at the slope of a hill which downhill a loading area with a rail way connection. The waste materials were formally transported by train to the nearby waste deposit in exploited brown coal open pit mines. We have conduct a previous study to analyze the transformation of HCH within annual cycles in trees using leaves, branches and fruits as well as soil and muck as substrates [4].

S2 Extraction and Clean-up of HCHs from Tree Trunks

The tree trunks were firstly separated into different parts based on the tree growth rings and frozen at −20 °C overnight and then freeze-dried in a freeze-dryer at −35°C and 0.310 mbar for 48 h. The bark samples are taken separately and up to 4-5 tree rings we combined as one section which are shown in Figure S1. The growth ring samples were taken with a wood driller as shown in Figure S2.

The wood cutting from drilling were powdered using a grinder (Retsch Ultrazentrifugalmühle, Retsch GmbH, Germany). Around 4-6 g frozen-dried plant tissue was extracted by ASE equipped with 22 mL stainless steel extraction cells. The extraction conditions were as follows: solvent: hexane/acetone (1:1, v:v); oven heat up time: 6 min; final temperature: 125 °C; static time: 3 min; pressure: 1500 psi; purge time: 60 s; flush volume: 60%. 3 consecutive static extraction cycles were performed and the extract combined. The extract of trunk tissues was transferred into a 50-mL round bottom flask, and the solvent was evaporated in a rotary evaporator at 40°C. Due to the low concentration of HCH in tree trunks, 5 repeats of one sample from a tree ring section of identical age were conducted using the extraction process above and the extracts were combined for further treatment. A small amount of DCM was added to re-dissolve the extracted materials. Then a small amount of Florisil was added to absorb the extracted materials and the DCM was evaporated to dryness for further clean-up.

A glass column (2.2 cm diameter × 32.5 cm length) was packed, from bottom to top, with glass wool as filter, 1 cm of clean sea sand, 15 cm of activated Florisil and 4 cm of activated anhydrous Na_2SO_4. Florisil was packed into column as slurry in hexane to ensure that the column is tightly and homogeneously packed. Prior to usage of the column, 15 mL hexane

was passed through the packed column. Then 100 μg of 7,12-dimethyl-benz[a]anthracene (2 mg mL^{-1} in hexane, 50 μL) was added on the top of the column as a UV-tracer. The extracted sample adsorbed to Florisil was then loaded onto column and eluted continuously by 30 mL hexane (1st fraction) and 50 mL hexane/DCM (v/v 1:1) (2nd fraction). The 1st fraction was discharged and the 2nd fraction containing quantitatively the HCHs was concentrated under a gentle nitrogen stream in a TurboVap concentrator. Then, the concentrated sample was transferred into a glass vial by a glass pipette and reconstituted into 1 mL using hexane for concentration and isotope analysis.

S3 Analytical Methods

Concentration Analysis. HCHs were separated with a HP-5 column (30 m × 320 µm × 0.25 µm, Agilent 19091J-413, USA) using helium as carrier gas with a flow of 2.2 mL min^{-1}. The oven temperature was held at 45 °C for 5 min, increased at 8 °C min^{-1} to 180 °C and then at 2 °C min^{-1} to 195 °C, finally increased at 8 °C min^{-1} to 220 °C and with a hold of 2 min. Samples were measured using splitless mode with an injector temperature of 250 °C. The injection volume was 1 µL and each sample was measured in triplicates.

Isotope Analysis. Carbon isotope composition. A Zebron ZB1 column (60 m × 0.32 mm × 1 µm; Phenomenex, Germany) operated with a constant carrier gas flow of 2 mL min^{-1} was applied for chromatographic separation. The oven temperature was initially held at 40 °C for 5 min, then increased at 10 °C min^{-1} to 175 °C followed by an increase at 2 °C min^{-1} to 200 °C with a hold of 10 min, and finally increased at 15 °C min^{-1} to 300 °C and held for 2 min. All samples were injected in splitless mode into a split/splitless injector hold at 250 °C. Each sample was analyzed in triplicate. The quality of the isotope data was controlled by analyzing a HCH standard with a known isotope composition, as described elsewhere [5].

Chlorine isotope composition. A gas chromatograph (Trace 1310, Thermo Fisher Scientific, Germany), equipped with an auto-sampler (TriPlus RSH, Thermo Fisher Scientific, Germany) was used for separation. Samples were injected in splitless mode with a constant carrier gas flow of 2 mL min^{-1}. Samples were separated on a Zebron ZB-1 capillary column using the same temperature program as used for the carbon isotope ratio measurement. Trichloroethene used as in-house reference compound (TCE2, $\delta^{37}Cl$ = -1.19‰) was added into the samples as an internal isotopic reference for identifying potential instrumental

drifts. The δ^{37}Cl was determined in two steps: (1) the deviation of a sample from TCE2 was calculated, and (2) raw δ^{37}Cl values were calibrated to SMOC scale by applying a two-point calibration approach. Calibration to SMOC scale was performed using in-house standards with confirmed off-line chlorine isotope composition, including methyl chloride (MeCl, δ^{37}Cl = +6.02‰) and trichloroethene (TCE2, δ^{37}Cl = -1.19‰). In addition, trichloroethene (TCE6, δ^{37}Cl = +2.17‰) and tetrachloroethene (PCE1, δ^{37}Cl = -0.55‰) were used for the validation of calibration, as described by Horst et al [6].

Enantiomer Analysis. Racemic compounds have an EF (-) equal to 0.5. An EF (-) > 0.5 indicates the preferential transformation of (+) enantiomer, and an EF (-) <0.5 indicates the preferential transformation of (-) enantiomer. [7] The oven temperature was initially held at 70 °C for 4 min, then increased at 10 °C min^{-1} to 120°C, at 1 °C min^{-1} to 169 °C, and finally increased at 10 °C min^{-1} to 220 °C and held for 8 min isothermally.

S4 Calculation of Biotransformation of HCH based on the Rayleigh Equation.

For the quantification of α-HCH transformation by isotope analysis, the simplified Rayleigh equation was applied for calculation biodegradation (B%), as shown in eq 1. δ_0 is the initial or referential carbon isotope values of the whole study and in current study δ_0 represents the carbon and chlorine isotope value of HCH-muck with a value of –27.2 ‰ and –1.9 ‰, respectively. Δδ is the difference of carbon isotope value of environmental samples between the HCH-muck. $\varepsilon_c/\varepsilon_{cl}$ are the carbon/chlorine isotope fractionation factors.

$$B\% = [1 - \left(\frac{\delta 0 + \Delta\delta + 1}{\delta 0 + 1}\right)^{1/\varepsilon}] \times 100 \quad (1)$$

S5 Calculation of Reconstructed Concentration of HCH in Different Sections of Tree Trunks.

The reconstructed concentration (RC) was calculated using the percentages of biodegradation and the residual concentration in different sections, which is shown in eq 2.

$$\mathrm{RC}(mg/kg) = \frac{\text{residual concentration } (mg/kg)}{(100 - B)\%} \quad (2)$$

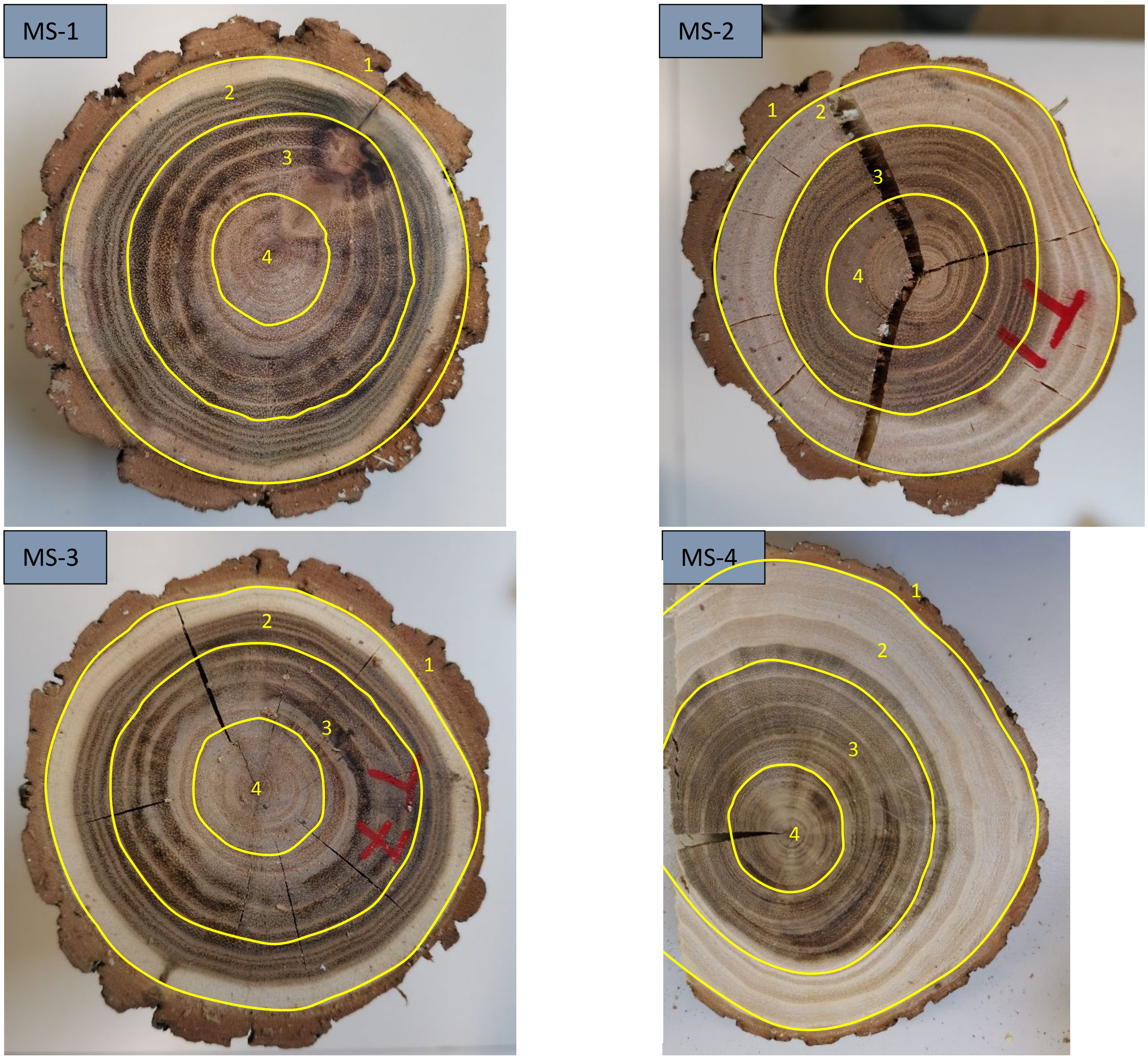

Figure S1. The sampling procedure of tree trunks based on the tree growth rings. Around 4 growth rings were defined as one sample. The number 1, 2 ,3 and 4 in the picture represents the bark sample, outer, inner and middle tree growth ring section, respectively.

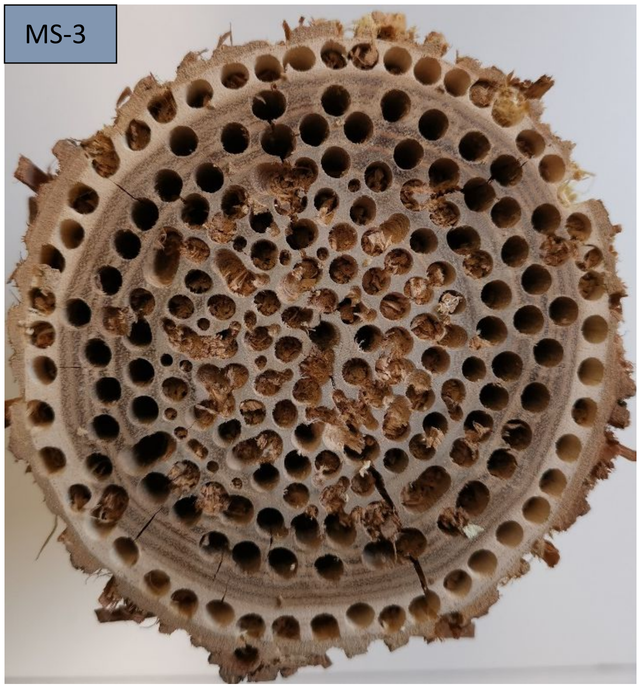

Figure S2. Example for the drilling procedure of tree trunks based on the tree growth rings.

Table S1. The different section of tree trunks with corresponding number for the age of the growth rings for each section.

	Bark	Outer section	Inner section	Middle section
T-1	0	1-5	6-9	10-13
T-2	0	1-4	5-9	10-14
T-3	0	1-5	6-9	10-14
T-4	0	1-5	6-9	10-13

Table S2. Biodegradation (%) in different sections of tree trunks

	Different sections of trunks	Calculation using carbon isotope fraction factors(ε_C)			Calculation using chlorine isotope fraction factors (ε_{Cl})	
		Aerobic	Anaerobic	LinA	Anaerobic	LinA
T-1	Bark	99.9->99.9%	99.8-95.2%	98.4-69.5%	37.3-62.3%	20.4-53.5%
	Outer	99.8->99.9%	91.9-99.5%	62.6-96.7%	32.3-55.8%	17.4-47.3%
	Inner	99.8->99.9%	92.7-99.6%	64.0-97.1%	38.8-64.2%	21.4-55.4%
	Middle	nc	nc	nc	45.6-72.0%	25.8-63.2%
T-2	Bark	99.9->99.9%	97.5-99.9%	76.4-99.3%	54.2-80.5%	31.8-72.3%
	Outer	99.9->99.9%	97.8-99.9%	77.4-99.4%	54.1-80.4%	31.7-72.2%
	Inner	99.8->99.9%	96.9-99.9%	74.3-99.1%	53.7-80.0%	31.4-71.7%
	Middle	85.7-99.0%	55.3-81.4%	26.9-66.3%	59.4-84.9%	35.7-77.3%
T-3	Bark	99.9->99.9%	92.3-99.6%	64.5-97.3%	45.4-71.7%	25.6-62.9%
	Outer	99.9->99.9%	97.1-99.9%	74.8-99.2%	55.1-81.3%	32.4-73.2%
	Inner	99.9->99.9%	93.0-99.6%	64.5-97.3%	44.8-71.2%	25.3-62.4%
	Middle	nc	nc	nc	54.9-81.1%	32.3-73.0%
T-4	Bark	99.4->99.9%	88.2-98.8%	64.5-97.3%	51.7-78.2%	30.0-69.8%
	Outer	99.9->99.9%	97.5-99.9%	74.8-99.2%	47.9-74.4%	27.3-65.7%
	Inner	99.2->99.9%	86.6-98.5%	64.5-97.3%	42.6-68.7%	23.8-59.9%
	Middle	nc	nc	nc	44.4-70.1%	24.9-61.8%

nc represents the values cannot be calculated due to no carbon isotope fractionation. >99.9% means that the calculation give unreasonable high values due to pathways with low isotope fractionation factors (ε).

Table S3. Reconstructed concentration (mg/kg) in different sections of tree trunks assuming biodegradation have changed the isotope composition in the various sections

Different sections of trunks		Calculation using carbon isotope fraction factors(ε_C)			Calculation using chlorine isotope fraction factors (ε_{Cl})	
		Aerobic	Anaerobic	LinA	Anaerobic	LinA
T-1	Bark	953.1–na	12.6-348.8	2.0-36.9	0.96-1.56	0.75-1.29
	Outer	28.5–na	0.8-12.4	0.2-1.9	0.09-0.14	0.08-0.12
	Inner	19.1–na	0.5-8.1	0.1-1.2	0.06-0.09	0.04-0.08
	Middle	nc	nc	nc	0.09-0.17	0.07-0.13
T-2	Bark	3182.2–na	16.4-936.5	1.7-60.6	0.88-2.06	0.59-1.45
	Outer	1002.1–na	4.4-284.5	0.4-17.1	0.21-0.50	0.14-0.35
	Inner	349.1–na	2.5-110.3	0.3-8.4	0.16-0.37	0.11-0.27
	Middle	0.4-5.2	0.1-0.3	0.1-0.2	0.12-0.33	0.08-0.22
T-3	Bark	242.5–na	5.6-100.9	1.1-14.2	0.71-1.38	0.52-1.05
	Outer	882.5–na	5.8-274.3	0.7-20.1	0.37-0.90	0.25-0.63
	Inner	79.3–na	1.8-33.0	0.4-4.6	0.23-0.44	0.17-0.34
	Middle	nc	nc	nc	0.20-0.48	0.13-0.33
T-4	Bark	66.1–na	3.2-32.7	0.9-6.8	0.78-1.73	0.54-1.25
	Outer	381.3–na	2.0-112.1	0.2-7.3	0.09-0.19	0.07-0.14
	Inner	9.5–na	0.6-4.9	0.2-1.1	0.13-0.24	0.10-0.18
	Middle	nc	nc	nc	0.11-0.21	0.08-0.16

na represents reconstructed concentration values which become equal to the weight of biomass which is unlikely. nc represents the values cannot be reconstructed due to no carbon isotope fractionation.

The reconstructed values of initial concentration calculated employing low fractionation factor which was found in aerobic pathway give unreasonably high initial concentration when the degradation exceed 99.99%. On one hand the

uncertainty in the calculation become unreasonable large with increasing difference of isotope values between source and sample ($\Delta^{13}C$). On the other hand, this may show that the isotope fractionation factor (ε) is not representative for the degradation in the trunk. Therefore, the reconstruction needs to be taken with caution.

Reference

(1) Kalbitz, K.; Wennrich, R. Mobilization of Heavy Metals and Arsenic in Polluted Wetland Soils and Its Dependence on Dissolved Organic Matter. *Sci. Total Environ.* **1998**, *209* (1), 27–39. https://doi.org/10.1016/S0048-9697(97)00302-1.

(2) Bunge, M.; Kähkönen, M. A.; Rämisch, W.; Opel, M.; Vogler, S.; Walkow, F.; Salkinoja-Salonen, M.; Lechner, U. Biological Activity in a Heavily Organohalogen-Contaminated River Sediment (8 Pp). *Environ. Sci. Pollut. Res.* **2007**, *14* (S1), 3–10. https://doi.org/10.1065/espr2006.03.298.

(3) Brack, W.; Kind, T.; Schrader, S.; Möder, M.; Schüürmann, G. Polychlorinated Naphthalenes in Sediments from the Industrial Region of Bitterfeld. *Environ. Pollut.* **2003**, *121* (1), 81–85. https://doi.org/10.1016/S0269-7491(02)00200-2.

(4) Liu, X.; Yang, A.; Richnow, H. H. Uptake and Metabolization of HCH Isomers in Trees Examined over an Annual Growth Period by Compound-Specific Isotope Analysis and Enantiomer Fractionation. *Environ. Sci. Technol.* **2022**, *56* (14), 10120–10130. https://doi.org/10.1021/acs.est.2c02697.

(5) Liu, Y.; Bashir, S.; Stollberg, R.; Trabitzsch, R.; Weiß, H.; Paschke, H.; Nijenhuis, I.; Richnow, H. H. Compound Specific and Enantioselective Stable Isotope Analysis as Tools to Monitor Transformation of Hexachlorocyclohexane (HCH) in a Complex Aquifer System. *Environ. Sci. Technol.* **2017**, *51* (16), 8909–8916. https://doi.org/10.1021/acs.est.6b05632.

(6) Horst, A.; Renpenning, J.; Richnow, H. H.; Gehre, M. Compound Specific Stable Chlorine Isotopic Analysis of Volatile Aliphatic Compounds Using Gas Chromatography

Hyphenated with Multiple Collector Inductively Coupled Plasma Mass Spectrometry. *Anal. Chem.* **2017**, *89*, 9131–9138. https://doi.org/10.1021/acs.analchem.7b01875.

(7) Liu, Y.; Bashir, S.; Stollberg, R.; Trabitzsch, R.; Weiß, H.; Paschke, H.; Nijenhuis, I.; Richnow, H. H. Compound Specific and Enantioselective Stable Isotope Analysis as Tools to Monitor Transformation of Hexachlorocyclohexane (HCH) in a Complex Aquifer System. *Environ. Sci. Technol.* **2017**, *51* (16), 8909–8916. https://doi.org/10.1021/acs.est.6b05632.
